# Corona Virus Disease 2019 (COVID-19) Presenting as Acute ST Elevation Myocardial Infarction

**DOI:** 10.7759/cureus.7782

**Published:** 2020-04-22

**Authors:** Suman Siddamreddy, Ramakrishna Thotakura, Vasuki Dandu, Sruthi Kanuru, Sreenath Meegada

**Affiliations:** 1 Internal Medicine, Baptist Health Medical Center, Little Rock, USA; 2 Nephrology, University of Arkansas for Medical Sciences, Little Rock, USA; 3 Neurology, Baptist Health Medical Center, Little Rock, USA; 4 Rheumatology, Central Arkansas Veterans Healthcare System, Little Rock, USA; 5 Rheumatology, University of Arkansas for Medical Sciences, Little Rock, USA; 6 Internal Medicine, The University of Texas Health Science Center/Christus Good Shepherd Medical Center, Longview, USA

**Keywords:** covid-19, stemi, acute respiratory distress syndrome, atypical covid

## Abstract

Patients with Covid-19 disease commonly present with symptoms related to respiratory illness, and less commonly they develop cardiovascular complications either on presentation or during the course of the disease. The mortality/morbidity is high in these patients with cardiovascular involvement. Acute ST-elevation myocardial infarction (STEMI) is a medical emergency which needs immediate coronary re-perfusion for better patient outcomes. Here we present a patient who presented to the emergency room with acute STEMI and later tested positive for COVID-19. She was successfully treated with coronary revascularization and stent placement, and remains on the ventilator to date as she quickly developed acute respiratory distress syndrome. We need more research in Covid-19 patients with cardiovascular involvement for early diagnosis, prevention of exposure to health care workers and effective treatment.

## Introduction

Severe acute respiratory syndrome-Corona virus-2 (SARS-CoV-2), a novel corona virus, has alarmed the health care communities across the world in a very short span of time posing an unprecedented challenge. Initial cases of this disease were reported in Hubei providence of China in December 2019 [1]. The World Health Organization named this disease as Corona Virus Disease 2019 (Covid-19) in February 2020. The patients with this disease usually present with features of respiratory illness. Rarely they have acute coronary syndrome on arrival. Our patient presented with acute ST-elevation myocardial infarction (STEMI) as initial presentation. To our knowledge, this is the first case of Covid-19 disease patient presenting with STEMI.

## Case presentation

A 61-year-old morbidly obese African American female presented to emergency department with chief complaint of left-sided chest pain of one-hour duration with progressively worsening shortness of breath and cough and some body aches for past few days. Her medical history was pertinent for coronary artery disease requiring multiple stent placements, chronic obstructive pulmonary disease, pulmonary hypertension, smoking dependence, uncontrolled diabetes mellitus, obstructive sleep apnea with continuous positive airway pressure use, cerebrovascular accident, and gastro-esophageal reflux disease. She denied any fever, sore throat and gastrointestinal symptoms. She did not have any exposure to sick contacts.

**Figure 1:**
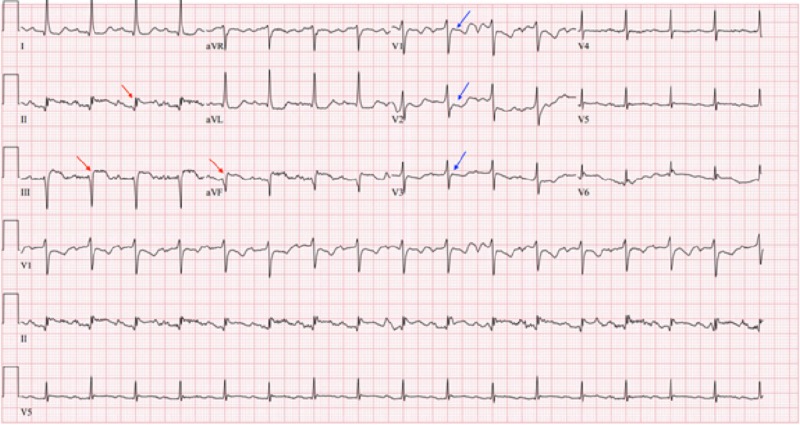
Electrocardiogram (EKG) showing ST elevation changes (Red arrow) in leads II, III and aVF. Reciprocal changes- ST depressions in leads V1, V2, V3 (Blue arrow)

Initial evaluation in the emergency room revealed temperature of 98.4 F, blood pressure of 146/84, respiratory rate in mid-30’s, heart rate of 99, and saturating 95% on 15 liters oxygen. Stat electrocardiogram (EKG) revealed ST elevations in leads II, III, aVF with reciprocal changes in V1, V2 consistent with acute inferior wall STEMI (Figure 1).

**Figure 2:**
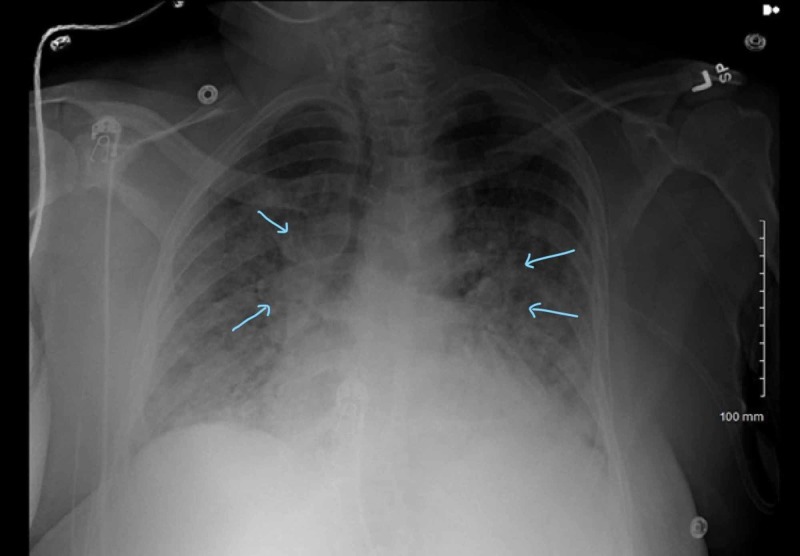
Chest X-ray suggestive of bilateral pulmonary edema, more on the right compared to left (arrows pointing)

Labs were significant for WBC of 9.8, elevated cardiac enzymes including CK of 606, CK-MB of 42, Troponin I of 3.53, elevated brain natriuretic peptide (BNP) of 4960, and elevated liver function tests including aspartate aminotransferase (AST) of 168 and alanine aminotransferase (ALT) of 93. Chest X-ray at that time showed diffuse bilateral pulmonary infiltrates consistent with cardiogenic edema (Figure 2).

**Figure 3:**
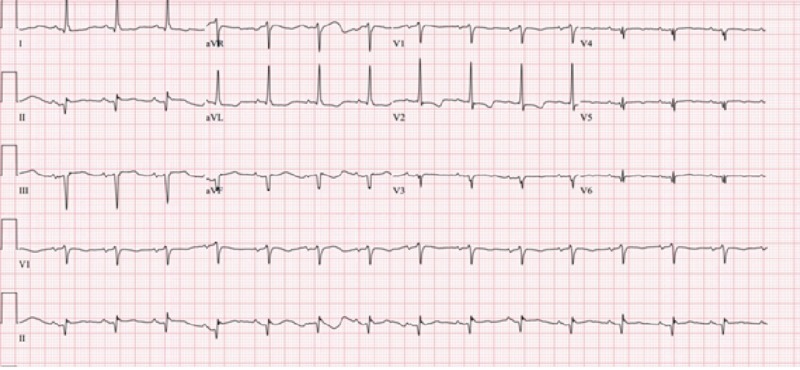
Electrocardiogram (EKG) showing resolution of ST elevation and reciprocal ST depression changes after the heart catheterization and stent placement

Cardiologist was notified immediately, and the patient was taken to the cardiac catheterization lab. Emergent left heart catheterization (LHC) showed subtotal occlusion of her previous stent located in right coronary artery (RCA). She was treated with aspiration thrombectomy and a drug eluting stent placement. Repeat EKG after the stent placement showed resolution of ST-elevation changes (Figure 3).

**Figure 4:**
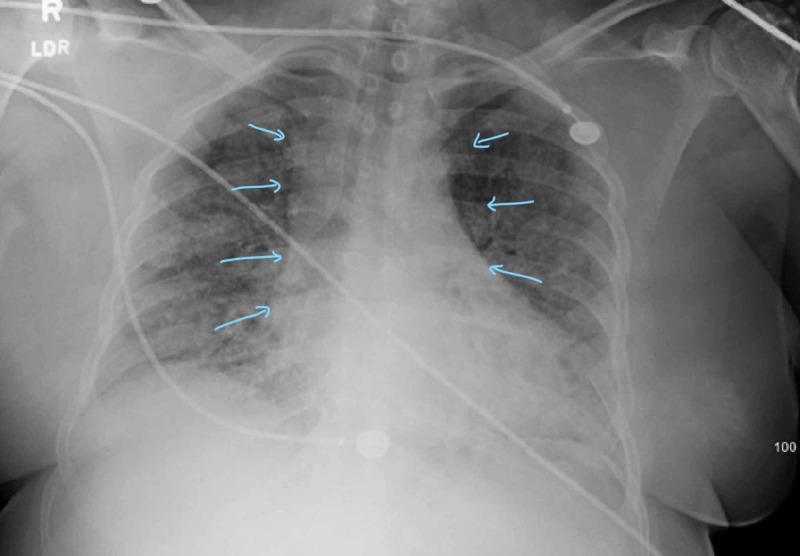
Chest X-ray showing bilateral ground glass opacities with worsening aeration suggestive of acute respiratory distress syndrome (ARDS) (arrows pointing)

Echocardiogram post LHC showed moderately reduced ejection fraction (EF) of 30-35%. Her previous echo from 2018 showed an EF of 65%. The patient was transferred to intensive care unit after the stent placement and was initially placed on bilevel positive airway pressure (BIPAP) followed by intubation due to worsening hypoxia. X-ray repeated at that time showed bilateral ground glass opacities more consistent acute respiratory distress syndrome (ARDS) (Figure 4).

Covid-19 test was sent out and it came back positive.

She was started on hydroxy-chloroquine along with azithromycin immediately and continued on mechanical ventilation with ARDS protocol. Her troponins peaked to 18 and then started to trend down. Labs done the next day showed elevated lactic acid dehydrogenase (LDH) of 2850, D-Dimer of 2000. Currently, the patient is on a ventilator with positive end expiratory pressure (PEEP) of 10, and 100% Fio2.

## Discussion

SARS-CoV-2 virus is one the seven corona viruses that causes infections in humans. The first case was identified in December 2019 in Wuhan, China [1]. In a very short period, it turned into a pandemic with 1,210,956 cases and 67,594 deaths reported from 178 countries as per the latest situation report-77 from the World Health Organization on April 6, 2020 [2]. Whenever a new infectious disease emerges, knowledge regarding clinical features, diagnostic tools, treatment options, and transmission dynamics is very critical. Patients with this disease, can transmit the virus even in asymptomatic phase. Most of the time, patients developed cardiovascular complications after the diagnosis of Covid-19 disease. Very rarely, patients presented with acute cardiovascular symptoms like our patient. Prior history of cardiovascular disease always poses poor outcomes with Covid-19 infection like any other respiratory infection. Studies have shown that Covid-19 infection can worsen existing cardiovascular problems or can precipitate new cardiovascular problems [3].

Pathogenesis

There were several mechanisms by which SARS-CoV-2 virus can cause cardiovascular complications. It can cause direct myocardial injury by altering angiotensin converting enzyme 2 signaling pathways as it binds to ACE2 receptors of myocardium and lung after entry into the human body [4,5]. It can also cause systemic inflammation and cytokine storm which can cause multi organ failure including cardiovascular involvement [6,7]. Hypoxia and respiratory failure pose a demand-supply mismatch causing myocardia injury. Prothrombotic state along with systemic infection causing shear stress from increased coronary flow can precipitate acute myocardial syndrome which is possibly the precipitating factor in our patient. Major electrolyte imbalances due to multiorgan failure and some drugs used for treatment of severe covid-19 infections have been shown to precipitate arrhythmias as well [8].

Clinical features

Common symptoms of Covid-19 have been reported as fever, cough, myalgias, headache and respiratory symptoms. Respiratory involvement can vary from flu-like illness, pneumonia to severe acute respiratory disease syndrome [7]. Cardiovascular symptoms and gastrointestinal symptoms have been reported from time to time. Mean incubation period was 5.2 days. There is a broad spectrum of cardiovascular-related symptoms that can occur in Covid-19 patients. They include exertional shortness of breath, orthopnea, chest pain, palpitations, and syncope. They can also present with or can develop acute myocardial syndrome, nonspecific myocardial injury, myocarditis, major arrhythmias, heart blocks and pulmonary embolism.

Most common cardiovascular complication in Covid-19 so far reported is acute myocardial injury which is defined as increase of troponin I above the 99th percentile in most of the studies. There are roughly 8-12% of cases reported that have shown significant troponin elevation [9]. Studies from SARS outbreak in the past showed that viral ribonucleic acid was present in cardiomyocytes during autopsies of some patients indicating direct myocardial involvement of the virus [10]. Diagnostic testing includes cardiac enzymes, echocardiogram, EKG, BNP, D-dimer and cardiac catheterization. We should be vigilant of all the possible cardiovascular complications to diagnose them early and treat them in a timely manner. At the same time, we should evaluate the risk-benefit ratio of ordering these tests, in patients where the management is not going to change much, because it puts the other health care workers at higher risk of exposure.

Treatment

Even though most of the patients with covid-19 infection present to the hospitals with primary respiratory symptoms, fever and myalgias, hospital systems should be vigilant about patients presenting with acute cardiovascular complications especially during this pandemic to minimize exposure to healthcare workers. Good history taking skill is still one of the vital steps in management of these patients. Hospitals should have well-defined policies calculating the risk-benefit ratio of each patient by considering percutaneous intervention vs. thrombolysis options. On top of this, all healthcare workers taking care of these patients should practice all precautions including appropriate use of personnel protective equipment according to the existing guidelines.

Lastly, we also need to be fully aware of the cardiovascular side effects of the drugs that are used to treat patients with Covid-19. Angiotensin converting enzyme inhibitors (ACEi) and angiotensin receptor blockers (ARBs) have been shown to upregulate ACE2 receptors in various cells including cardiomyocytes. Even though there were theories that the use of these medications can up-regulate receptors and cause worsening disease symptoms, there were no experimental or clinical data to support this. So many professional organizations like American College of Cardiology (ACC) and American Heart Association (AHA) recommended not to stop those medications in patients affected with Covid-19 disease, especially if they do not have any other contraindications [11].

An open label non-randomized trial showed that azithromycin and hydroxychloroquine are potential therapeutic options in patients with the disease [12]. These drugs can cause QT prolongation and severe ventricular arrhythmias. So, we need to be cautious when we use these medications by closely monitoring their QT interval and try to avoid other concomitant medications that can prolong the QT interval. We need more research to look into the cardiovascular problems in Covid-19 patients to appropriately treat them with minimal complications.

## Conclusions

Patients with Covid-19 usually present with respiratory-related problems along with fever, chills and myalgias. Rarely they can present with symptoms related to cardiovascular disease or can have worsening cardiovascular symptoms during the course of the disease. Some patients like our patient, who presented with atypical clinical characteristics, pose a challenge. So, in the current pandemic situation, we recommend the front-line physicians to be vigilant about atypical presentations of Covid-19 disease and diagnose them early and treat them appropriately.

## References

[1] Lake MA (2020). What we know so far: COVID-19 current clinical knowledge and research. Clin Med (Lond).

[2] (2020). Coronavirus disease 2019 (COVID-19): Situation report - 77. disease.

[3] Bansal M (2020). Cardiovascular disease and COVID-19. Diabetes Metab Syndr.

[4] Xiong TY, Redwood S, Prendergast B, Chen M (2020). Coronaviruses and the cardiovascular system: acute and long-term implications. Eur Heart J.

[5] Li B, Yang J, Zhao F (2020). Prevalence and impact of cardiovascular metabolic diseases on COVID-19 in China. Clin Res Cardiol.

[6] Zhou F, Yu T, Du R (2020). Clinical course and risk factors for mortality of adult inpatients with COVID-19 in Wuhan, China: a retrospective cohort study. Lancet.

[7] Huang C, Wang Y, Li X (2020). Clinical features of patients infected with 2019 novel coronavirus in Wuhan, China. Lancet.

[8] Driggin E, Madhavan MV, Bikdeli B (2020). Cardiovascular considerations for patients, health care workers, and health systems during the coronavirus disease 2019 (COVID-19) pandemic. J Am Coll Cardiol.

[9] Lippi G, Plebani M (2020). Laboratory abnormalities in patients with COVID-2019 infection. Clin Chem Lab Med.

[10] Oudit GY, Kassiri Z, Jiang C, Liu PP, Poutanen SM, Penninger JM, Butany J (2009). SARS-coronavirus modulation of myocardial ACE2 expression and inflammation in patients with SARS. Eur J Clin Invest.

[11] (2020). HFSA/ACC/AHA statement addresses concerns Re: using RAAS antagonists in COVID-19. https://www.acc.org/latest-in-cardiology/articles/2020/03/17/08/59/hfsa-acc-aha-statement-addresses-concerns-re-using-raas-antagonists-in-covid-19.

[12] Gautret P, Lagier JC, Parola P (2020). Hydroxychloroquine and azithromycin as a treatment of COVID-19: results of an open-label non-randomized clinical trial. Int J Antimicrob Agents.

